# Experiences of goal planning in Australian community pharmacy settings for people experiencing mental illness: A qualitative study

**DOI:** 10.1111/hex.13818

**Published:** 2023-07-10

**Authors:** Victoria Stewart, Sara S. McMillan, Jie Hu, Jack C. Collins, Sarira El‐Den, Claire O'Reilly, Amanda J. Wheeler

**Affiliations:** ^1^ Centre for Mental Health Griffith University Nathan Australia; ^2^ Menzies Health Institute Queensland Griffith University Nathan Australia; ^3^ School of Pharmacy and Medical Sciences Griffith University Nathan Australia; ^4^ Faculty of Medicine and Health The University of Sydney School of Pharmacy, The University of Sydney Camperdown New South Wales Australia; ^5^ School of Pharmacy, Faculty of Health and Behavioural Sciences University of Auckland Auckland New Zealand

**Keywords:** community pharmacy, goal planning, goal setting, severe and persistent mental illnesses

## Abstract

**Background:**

Person‐centred goal planning is increasingly being incorporated into healthcare interventions. People experiencing severe and persistent mental illnesses (SPMIs) have high levels of co‐occurring health conditions, reducing their life expectancy when compared with the general population. As medications are commonly used in the treatment of SPMIs, community pharmacists are well‐placed to support the health and wellbeing of this population.

**Objectives:**

To examine pharmacists' and service users' experiences of goal planning as a component of a community pharmacy‐based health intervention for people experiencing SPMIs (*PharMIbridge* intervention).

**Methods:**

This study utilised a qualitative exploratory approach with an interpretive description method. Semistructured interviews were undertaken with community pharmacists (*n* = 16) and service user participants (*n* = 26) who had participated in pharmacist support services for people experiencing SPMIs (*PharMIbridge* intervention*)*.

**Results:**

Four themes relating to goal planning were identified. First, goal planning provided purpose and motivation for participation in the intervention. Planning realistic goals was important but often challenging. Both pharmacists and service users highlighted the relational aspects of goal planning and how strong relationships supported positive behaviour change and outcomes. Finally, individualised and flexible approaches were important aspects of the intervention, ensuring goals were meaningful to service users.

**Conclusions:**

The findings from this study identified positive outcomes from the inclusion of goal‐planning processes in a community pharmacy‐based health intervention. Further research regarding tools, strategies or training that could support future goal‐planning interventions in primary healthcare is needed.

**Patient or Public Contribution:**

The *PharMIbridge* randomised controlled trial research team included lived experience members and was overseen by an expert panel that included members with a lived experience of mental illness and representatives from key organisations. The training provided to pharmacists was co‐designed and co‐delivered by the researchers and lived experience representatives, and pharmacists were supported by lived experience mentors. Service user participants were invited to participate in the interviews through a number of pathways (e.g., at the completion of the intervention, flyers). Those interested were provided with the full study participant information and provided with a $30 gift voucher at the conclusion of the interview.

## 1 INTRODUCTION

Goal planning is commonly used in rehabilitation, physical and mental healthcare settings to promote behaviour change, providing a focus for health interventions and a sense of direction for all involved.^1^ Goal planning is generally understood as the process of discussion and negotiation through which health practitioners and service users identify priorities for treatment and plan how best to meet desired outcomes.^2^ A number of psychological theories have influenced the development of goal planning, including the self‐efficacy components of social cognitive theory^3^; internal factors (e.g., motivation) in goal‐directed behaviour identified within goal setting theory^4^ and the health action process approach.^5^ These theories suggest that goal planning influences behaviour by engaging service users in their treatment, activating motivation, directing effort and promoting problem‐solving to overcome difficulties.^6^, ^7^ In addition, several mechanisms through which goal planning supports positive outcomes have been identified, including increasing service user motivation, team communication, service user ownership and specificity of treatment planning.^8^

Person‐centred care, where service users identify and set their own health goals, is increasingly recognised as a guiding principle in healthcare.^9^ Increased involvement of service users in the co‐production of their healthcare has resulted in enhanced care experiences, improved health outcomes and lowered healthcare costs.^10^ In addition, improved physical and psychological health status and increased capacity to self‐manage illnesses have been identified as important outcomes of person‐centred care for people experiencing long‐term health conditions.^11^ Within mental healthcare, person‐centred approaches have resulted in increased engagement in treatment, improved medication adherence and wellbeing and reduced symptom severity.^12^ Community pharmacists are increasingly incorporating person‐centred approaches when delivering interventions for service users experiencing long‐term health conditions or interventions aimed at improving health (e.g., smoking cessation, weight management, sexual health).^13^

People experiencing severe and persistent mental illnesses (SPMIs), including schizophrenia and other psychotic disorders, bipolar disorder and severe and recurrent depression, experience high levels of personal, social and economic impacts.^14^ In addition, these individuals have higher rates of physical health concerns such as respiratory illnesses, obesity, diabetes and cardiovascular disease, reducing their life expectancy by 10–25 years.^15^ As such, policymakers have prioritised providing appropriate person‐centred, coordinated support for this population.^16^, ^17^, ^18^

As many people experiencing SPMIs have co‐occurring health conditions, complex medication regimes with significant side effect profiles and poor treatment adherence,^19^ community pharmacists are well‐placed to provide a range of person‐centred support and interventions for this population. In addition, community pharmacists are skilled in delivering medication‐related information and advice, promoting safe and effective medication use and encouraging healthy lifestyle behaviours.^14^ The Bridging the Gap between Physical and Mental Illness in Community Pharmacy *(PharMIbridge)* randomised controlled trial (RCT) was designed to address key healthcare gaps for this population through an individualised, goal‐directed intervention in community pharmacy settings.^20^ The intervention required pharmacists and service users to work together to identify areas of need or personal concern and co‐develop goals and an activity plan to support the achievement of these goals. A total of 512 goals were planned during the *PharMIbridge* intervention with 53.7% of goals related to lifestyle changes (e.g., exercise, weight), 15% concerning the management of physical health concerns (e.g., review of current treatment, strategies to improve health), 12.1% regarding the use of medicines (e.g., adherence strategies, safety and tolerance), 9.6% the management of mental health conditions (e.g., psychoeducation, referrals to treating practitioners) and 9.6% improving satisfaction with life (e.g., relationships, employment, leisure).

Goal planning research has overwhelmingly used quantitative methodologies to investigate the role of goals in gaining positive outcomes and improving performance.^21^ While measuring objective changes or outcomes has been important in understanding how goals can influence performance, limited literature has explored how goal planning strategies are incorporated into mental health practice and how people experience goal planning, particularly within community pharmacy settings. As such, this study used a qualitative approach to examine the experiences of goal planning from the perspective of the health practitioner (community pharmacists) and service users experiencing SPMIs.

## 2 METHODS

This study was conducted according to the Consolidated Criteria for Reporting Qualitative Research^22^ and the Standards for Reporting Qualitative Research.^23^ An exploratory study using an interpretive description research method was used to investigate and expand on current understandings of the experience of goal planning.^24^ Developed within the discipline of nursing, interpretive description is suited to investigating applied concepts and highlighting themes and patterns within research participants' subjective experiences to generate findings applicable to healthcare practice.^25^ Additionally, interpretive description recognises that knowledge and meaning are created from the interplay between the researcher and participants and stresses that findings must be grounded in the data.^26^ Ethical clearance was obtained from a University Human Research Ethics Committee (HREC/2019/473).

### 2.1 Settings

This study utilised data collected during the evaluation of the *PharMIbridge* RCT.^20^ The *PharMIbridge* RCT was undertaken between 2020 and 2022, recruiting community pharmacies (*n* = 51) in rural and urban settings across four regions of Australia. Pharmacies were randomly assigned to the *PharMIbridge* intervention group (IG) or comparator group (CG), which provided usual care in the form of a standard in‐pharmacy medication review service (MedsCheck: A MedsCheck is an Australian remunerated service whereby a community pharmacist reviews a person's medication, identifies and addresses any medication‐related problems [MRPs], and provides medication information. Australian residents are eligible for a MedsCheck service including consumers who are at a higher risk of MRPs, such as those using more than five prescription medicines^27^). The *PharMIbridge* intervention was an in‐depth support service provided by trained community pharmacists that included a holistic review and management of the service user's health concerns, particularly related to physical health and MRPs. To prepare pharmacy staff to participate in the RCT, both IG and CG pharmacy staff completed the *Blended‐Mental Health First Aid (MHFA) for the pharmacy* ^28^ course, with IG pharmacy staff (*n* = 55) receiving further training to support the provision of a co‐designed, individualised, support service for service users experiencing SPMIs. The training utilised case scenarios and role play to support the application of theory into practice,^29^ with further details described elsewhere.^30^, ^31^

The training consisted of four modules addressing the mind–body interface; complex psychotropic medication use; managing physical health concerns; communication, motivational interviewing and goal planning. It aimed to upskill pharmacy staff to confidently provide the *PharMIbridge* intervention. As such, the goal training module focused on the transtheoretical stages of change model,^32^ the Capability, Opportunity, Motivation to change Behaviour model for behaviour change,^33^ motivational interviewing^34^ and how to develop SMART (specific, measurable, achievable, realistic and time‐bound) goals.^35^

After the training, *PharMIbridge* IG pharmacy staff identified and recruited service users aged 16 years or over, who had been using antipsychotic or mood stabiliser medication(s) for SPMIs (for the purposes of the *PharMIbridge* RCT, SPMIs refer to any mental illnesses that had a continuous and significant effect on a person's daily life and included [but was not limited to] the following: schizophrenia and other psychotic disorders, severe and recurrent depression, bipolar disorder) regularly for the 6 months before recruitment, and who had unmet physical health concerns, were at risk of or already experiencing MRPs or both. Service user participants (*n* = 156) completed an initial health review with their community pharmacist, which involved a range of clinical measures (e.g., weight, blood pressure) and a range of self‐assessment questionnaires and tools aimed at recognising health and lifestyle concerns.^20^ Service users and pharmacists then identified key concerns regarding physical and psychological wellbeing and medication use before co‐designing individualised goals and goal plans. Pharmacists followed up with service user participants regularly over the next 6 months to review goals and adjust or create goals as required.

### 2.2 Recruitment and participants

At RCT completion, a criterion sampling approach was undertaken with all IG pharmacists and service users invited to participate in an interview. IG pharmacists were invited, by email, and an information sheet was provided, consent was sought and interested pharmacists were asked to contact the research team. A follow‐up email and phone calls were made to pharmacists who did not respond to the initial email invitation. Additionally, IG service users were invited to participate in an individual phone interview, with the information provided in the final health review and/or through flyers provided by their pharmacist. Service users were asked to contact the research team and were offered further information, had any questions answered and a suitable time to conduct the phone interviews was arranged. Of the pharmacists contacted, 29% agreed to participate and 16.7% of service users responded. Those who did not participate either did not respond to the invitations or stated they were too busy. The interviews were part of the final evaluation of the *PharMIbridge* intervention, discussing a number of aspects of participants' experiences. For the purpose of this study, the sections of the interview focused on goal planning were utilised. All interviews were undertaken by one author (V. S.) who had limited involvement in the intervention training or implementation. Interviews were completed between May 2021 and May 2022. V. S. is a female occupational therapist, qualitative researcher and mental health practitioner who has used goals extensively in clinical practice and is completing a PhD focused on goal planning.

Field notes were written after each interview and circulated to the research team. All interviews were audio recorded and transcribed by a third‐party transcription company. All participants were offered a copy of the transcript to ensure accuracy, with 11 participants requesting a copy, with no changes required. Transcripts were quality checked by the interviewer for accuracy. Service users were provided with an AU$30 gift voucher, and pharmacists were offered an AU$50 gift voucher on completion of the interview, accounting for the differences in the anticipated length of interview time.

A semistructured interview guide was developed based on a review of previous similar studies and then discussed by the research team. A service user mentor also reviewed the service user interview guide to ensure questions were appropriately worded and targeted. Goal planning‐focused questions were concentrated on the experience and process of goal planning, how goals were decided, the value of goal planning within the *PharMIbridge* intervention, barriers/facilitators to goal planning and achievement, and the experience of the pharmacist/service user relationship when planning goals (Table 1).

**Table 1:** Interview guides.

Service user goal planning focused interview questions
Can you describe the areas of your life that you and the pharmacist focused on? (e.g., medication, mental health, physical health issues)
Can you please describe the type of goals that you set with the pharmacist? What was the goal setting process like? (e.g., Have you set goals before? Who with? How were the goals documented? Did you have a copy of your goals and plan?) How were the goals decided? How did you decide that they were the right goals for you? What was the process of deciding how you would achieve your goals like? How did you decide the steps to take in achieving your goal? Were there any barriers to achieving your goals? What strategies did you discuss in addressing the barriers to achieving your goals? Were all your goals considered? How valuable was the goal setting aspect of the *PharMIbridge* service? Why/why not? What was the role of goal setting in supporting you to make changes to your life? Was it difficult/easy to achieve your goals? Why? Do you feel your participation in *PharMIbridge* helped you achieve your health goals?
What did you like most about the *PharMIbridge* service?
What did you like least about the *PharMIbridge service*?
Pharmacist goal planning focused interview questions
How did the *PharMIbridge* service work in the pharmacy to support people living with severe and persistent mental illness?
Can you please describe the **key impacts** of the *PharMIbridge* service for service users? Can you describe the goal‐setting process? How easy/difficult was it for service users to identify goals? How did you help service users decide on goals? What sort of goals did you set with service users e.g. lifestyle, medication? What was your experience of writing goals with service users? Did you feel confident to set goals with service users? What helped? Did you find the SMART goal‐setting process (discussed in the training) helpful? Did you think that the goal‐setting process was a useful part of the *PharMIbridge* service? Why/why not?
How easy or difficult did you find the process of follow‐up? Why?
Were there any challenges to delivering the *PharMIbridge* service?
How did the *PharMIbridge* service effect your role or practice as a pharmacist?
Now that the *PharMIbridge* RCT is finished, is there any aspect/s of the service that you will continue to integrate into your practice in the future?
Is there anything else you would like to share about your experience in the *PharMIbridge* RCT?

Abbreviations: RCT, randomised controlled trial; SMART, specific, measurable, achievable, realistic and time‐bound.

### 2.3 Data analysis

Data analysis occurred after data collection was completed. The availability of respondents determined the sample size; however, repetition of concepts or themes occurred as interviews progressed with no new themes generated, providing confidence that the sample size was adequate. The data analysis and collection occurred concurrently, and pharmacist and service user interviews were analysed together. This allowed for some flexibility to deviate from the interview guide as data collection progressed to provide further depth of discussion to reflect an evolving understanding of the experience of goal planning. In accordance with interpretive description approaches,^24^ interview data were analysed using the constant comparative method.^36^ Transcripts were independently read by two authors (V. S., S. M.), describing and documenting key aspects of the data and identifying patterns, themes and novel topics related to the experience of goal planning. S. M. is a community pharmacy academic with a strong background in qualitative research. An inductive development of themes was undertaken by one author (V. S.), grouping similar codes into broader categories within NVivo software^37^ and comparing commonalities and variations across both data sets.^38^ The two authors involved in the analysis met regularly to discuss the synthesis of data, refining themes and reflecting on how their individual professional backgrounds and experiences influenced the interpretation and portrayal of the data. No member checking of the analysis was undertaken.

## 3 RESULTS

### 3.1 Participants' characteristics

Sixteen pharmacists and 26 service users were interviewed. At the time of the interviews, pharmacists were aged between 23 and 54 years (mean [*M*] = 36.4 years) and had an average of 12.5 years of pharmacy experience. The service user participants were primarily male (*n* = 16), aged between 34 and 71 years (*M* = 51.6 years), and taking medication for SPMIs for an average of 18.7 years. Pharmacist interviews lasted 35–75 min (*M* = 52 min), and service user interviews ranged from 14 to 48 min (*M* = 28.5 min). Table 2 presents further participant characteristics.

**Table 2:** Interview participant characteristics.

Characteristics		Number	Percentage
*Pharmacists* (*n* =16)			
Gender	Male	8	50
	Female	8	50
Age at interview, years	<30	6	37.5
	30–39	3	18.7
	40–49	6	37.5
	50–59	1	6.3
Role	Pharmacist	4	25
	Senior pharmacist or pharmacist‐in‐charge	6	37.5
	Pharmacy owner	6	37.5
Pharmacy experience, years	1–9	6	37.5
	19	6	37.5
	>20	4	25
*Service users* (*n* =26)			
Gender	Male	16	61.5
	Female	10	38.5
Age at interview, years	<40	6	23.1
	40–49	5	19.2
	50–59	8	30.8
	>59	7	26.9
Self‐reported primary mental health diagnosis	Schizophrenia	11	42.3
	Bipolar disorder	7	26.9
	Schizoaffective disorder	1	3.85
	Posttraumatic stress disorder	4	15.4
	Borderline personality disorder	1	3.85
	Unsure	2	7.7

### 3.2 Themes

Participants' descriptions of their experience highlighted four important themes relating to goal planning: (i) providing purpose and motivation; (ii) planning realistic goals; (iii) the relational aspects of goal planning and (iv) individualised and flexible approaches. These themes are discussed in further detail below, supported by quotations. Individual identifiers are provided to distinguish voices, with ‘SU’ designating service user voices and ‘P’ designating pharmacist voices.

### 3.3 Providing purpose and motivation

The majority of participants reported that goal planning provided a purpose for getting together and participating in the intervention. Goals were seen to give the intervention a structured approach, providing a focus for conversations. Some service users enjoyed the challenge of doing something new, while others described additional benefits that goal planning provided in establishing routines and distractions in their lives.

I think when managing mental health, having goals gives me something else to focus on instead of always worrying about all the things I have to manage with my illness and all these appointments I'm always at. And it helps me focus on something else as well. Which is a good thing. (SU12)

Several service user participants identified that participating in goal planning allowed them to reflect on their personal strengths and limitations, highlighting their priorities and plans for the future. In addition, working with the pharmacists to plan goals provided ‘a sense of clarity’ (SU7) and supported service users to attend to issues that had been ‘put off’ or placed in the ‘too hard basket’ (SU24).

It was because it made me stop and think that I had some goals, really. Before that, I really didn't think I had any goals. Like I didn't really speak them out in my mind, but it made me speak them out. It shone a light on what I should be doing. (SU5)

All service user participants identified the role that goal planning provided in motivating them to make changes to achieve their intended outcomes. Participants articulated that goal planning held them accountable for making changes, as they tried not to disappoint anyone. Regular reviews allowed them to track their progress, and several service users reported the importance of documenting goals on paper or on their phones to support motivation and tracking.

… knowing that you were going to be reporting to somebody in a couple of weeks made you sort of keep track of things better where I think at the moment I've got a tendency to let things lapse until I'm feeling a bit better. And feeling a bit better doesn't always happen. (SU24)

Service user motivation levels, past experiences of goal planning and confidence were articulated as impacting readiness to participate in goal planning discussions. Some service users reported that goal planning was initially daunting and felt uncomfortable committing to their goals. However, most stated that it became easier as the programme progressed, with routines becoming established and results from their efforts apparent.

At first, it seemed a bit tough … seemed like an impossible thing. And the losing weight, well as soon as I started the program, now I've lost like six and a half, seven kilos. As I got into it more, it just got easier and easier. Now, it's just a daily routine I have. (SU19)

Pharmacists noted that several service users had difficulty with goal planning due to the impacts of their mental illnesses and co‐occurring health conditions. Service users described barriers that impacted their experience of planning and achieving their goals. These included episodes of ill health, changes in personal circumstances, and COVID‐19 lockdowns affecting their routines, relationships and access to community and health supports. For example, some found the process overwhelming, as they were focused on ‘just living day‐to‐day’ (P13), managing appointments and coping with symptoms. Others, particularly those experiencing symptoms of depression, struggled to think aspirationally or ‘come up with anything that they would want’ (P4). However, positive encouragement and focusing on small achievements supported service users in maintaining their motivation.

[Goal planning] sets accountability, and it gives people a sense of accomplishment in what they're doing, so they do feel good once they've done it, and they find it very rewarding. (P13)

### 3.4 Planning realistic goals

Framing goals in small, realistic and achievable steps was essential in supporting service users to make changes. Each goal needed to be tailored to match the service user's abilities and time available to commit to action. Understanding what was important to each service user was crucial in setting goals that were realistic.

Just little goals, I think, are what's important to people in a distressful situation. That's all they can handle at that time. You can't give them massive goals. (P15)

Both pharmacists and service users described a process of collaborative brainstorming of ideas about how best to achieve goals. Having all the necessary information and knowledge available to assist people in identifying the most appropriate actions to meet their needs was considered significant. Pharmacists often used their professional knowledge to provide further information or searched for appropriate resources to provide to service users to help them understand their options.

So, you get a lot of general advice from different people, but because [the pharmacist] had a background in psychotropic medication, they were able to actually say it will be over at this time. I trusted them, and they were right, so having that kind of faith in them and their abilities really helped me anticipate withdrawal effects and outcomes and timelines. (SU26)

Pharmacists identified that it was often challenging to pinpoint realistic goals as many of the service users' issues were complex, with interacting elements and needs. For example, service users identified goals to lose weight, which could be related to several factors such as medication use, diet, exercise, or sleep. Understanding the service user's behaviours and unravelling the best course of action was often challenging. Additionally, many service users had complex physical and mental health conditions impacting their ability to achieve their goals, reinforcing the need to identify small, realistic changes.

No, one in particular still has significant weight concerns, medication‐related adverse effects, comorbidities of hypertension, diabetes, and it was simply creating some momentum to substitute things like iced coffees and soft drinks … basically saying all right if we can make this one change and get something moving. So it wasn't all addressed at once, and I don't think it would have been terribly successful if we tried to solve a lot of problems in the first consult. (P2)

### 3.5 The relational aspects of goal planning

Conversations about goals and progress towards achieving goals were critical touchpoints for ongoing contact and the development of relationships. Goal‐centred discussions were used as an ‘icebreaker’ (P4), often leading to further discussions about other things happening in people's lives and how they were managing daily tasks. Through these conversations, relationships deepened as pharmacists and service users learnt about each other. Service users highlighted the importance of having a health practitioner who listened, cared and ‘saw them as a person, rather than a name or number’ (SU2).

I've enjoyed every meeting that I've had with [the pharmacist], and I'd like to say, it certainly has brought us closer together where he has a better understanding of me and particularly my illness. (SU22)

Service users also described longer‐term outcomes from these strengthened relationships, subsequently describing their pharmacist as a trusted member of their healthcare team. Pharmacists also appreciated the opportunity to learn from service users about their lived experience of SPMIs, resulting in an increased understanding of some of the unique challenges experienced by service users. Pharmacists identified positive outcomes from investing time in relationship building and reported incorporating a larger focus on strengthening relationships with service users in the future.

So now, moving forward, the time that I've spent with these clients, the interactions that I had, the understanding, the insights that I've gained from these people has obviously impacted my practice and my understanding and knowledge to then help others in a similar way. (P8)

Focusing on relationship building was also seen as important in supporting service users who were not ready to take action or had experienced setbacks in their goal‐directed activity. Engaging in ongoing supportive relationships allowed people time to think about their options and easily re‐engage with goal planning once they felt ready and able.

Maybe that's all they need at that time, in their point in life. Maybe down the track, they might think about our conversation, and maybe they are ready. (P10)

That little bit more of a personal touch and for them to know what's going on and perhaps approach you with something down the track that you might not have thought of. (SU24)

Pharmacists noted that the *MHFA course* assisted them in confidently working with people experiencing distress and continuing to develop supportive relationships with them. These relationships and understanding of SPMIs were important outcomes from the goal planning process as well as ensuring that goals were owned by service users and appropriate to individual circumstances.

I'm more educated. I'm more understanding. I have a far better understanding of you know, if someone comes in and they're a bit short or a bit blunt or they've lost all their things or, you know, just having a much better understanding of what might be actually going on for them. And just being more willing to support them, as well. (P16)

### 3.6 Individualised and flexible processes

Several service users reported that they had not previously participated in goal planning and required knowledge and guidance to take part in the intervention. Goals were not always achieved within the 6‐month timeframe of the *PharMIbridge* intervention. Both pharmacists and service users expressed a desire for the intervention to continue for a longer period for relationship building and demonstrating the longer‐term outcomes of the lifestyle changes that were undertaken.

So yeah, I think that's probably why I think, in a lot of cases, it will take longer than a six‐month program to get there because some of them took a long time to warm up. (P2)

While the intervention had ceased, several service users reported that they were continuing to work towards their goals.

It's going to take time the rest of it, the goals are going to take time to get going. (SU2)

There was significant variability in the goals prioritised and planned by service users. As such, pharmacists reported that they needed to learn about resources in their local communities to refer service users to assist with goal achievement. Pharmacists identified a range of health practitioners and local supports that they engaged with as part of the intervention, including dentists, general practitioners, nurses, case managers, support workers, social workers, dieticians, podiatrists and physiotherapists, as well as community agencies and support groups such as Men's Shed, Pilates instructors, gyms and pools. Pharmacists also noted that they often took on an advocacy role, assisting service users in accessing resources in a timely manner.

So it just goes to show I guess those kind of interventions and the collaboration between different health professionals definitely has a role to play in the holistic health of the patient. So that was really nice to watch. (P13)

Flexibility in the goal planning and follow‐up processes was seen as essential to cater to the diverse needs of service users and the complexity of many of the presenting issues. Both service users and pharmacists appreciated the person‐centred approach and ability to respond to needs individually and creatively.

PharMIbridge was very flexible in where we go because every client is unique, and every need is unique. So that was good that it was unstructured in that we could explore things as they arose. (P8)

It gives you some support to bounce ideas off, and talk to people about what's going on, so you don't feel alone, or just out there by yourself. (SU11)

## 4 DISCUSSION

To our knowledge, this is the first study investigating the experience of goal planning with people experiencing SPMIs in a community pharmacy setting. The four themes describe and examine the processes of goal planning and the complexities of implementing goal planning in a community pharmacy setting for people experiencing SPMIs. For the most part, service user experiences aligned with many aspects of goal planning theory,^39^ including that goals motivated people to enact changes through increasing attention and effort, and progress was moderated by feedback, commitment, knowledge and ownership. Study findings also highlighted that person‐centred goal planning is an essential element in supporting behavioural change for people experiencing SPMIs and can potentially improve the health of service users.^40^

Participative approaches to goal planning were significant, with goal planning providing both a purpose to relationship building and a means to enhance relationships. Pharmacists highlighted the importance of learning about service users and their lived experiences of SPMIs, confirming previous research that found improved outcomes result from goal‐planning interventions that develop strong working alliances and listen to service user priorities.^41^, ^42^ Additionally, focusing on relationship development supported a collaborative, person‐centred approach to goal planning. Collaboration was seen to increase commitment to the goal and action plan. At the same time, the provision of ongoing support provided opportunities for feedback and tracking of progress, which is known to be an important moderator for goal achievement.^39^ Findings from this study suggest that future community pharmacy goal‐planning interventions should be developed in a manner that allows for adequate time for pharmacists to develop supportive relationships and provide ongoing support to maximise outcomes from goal planning. Additionally, given the importance of relationship development in the goal‐planning process, pharmacists would benefit from opportunities to expand interpersonal skills and confidence to work alongside service users experiencing SPMIs.

While person‐centred approaches are recommended for goal planning,^43^ the results from this study indicated that a number of service users felt unprepared to participate in goal planning discussions. A range of issues impacted service user readiness to participate in goal planning discussions (e.g., motivation levels, confidence, past experiences of goal planning). Some pharmacists reported providing participants with information and resources to assist in their decision‐making, aligning with previous research that found providing information supported informed decision‐making and goal negotiation.^44^, ^45^ Pharmacists also noted that person‐centred goal planning was not always a straightforward process and was influenced by the complexity of service user needs. Additionally, simply asking service users to articulate their goals has been found to be problematic, as many are unaware of what goals might be appropriate in new contexts.^46^ As such, developing and providing tools that explain goal planning to prepare service users to participate in goal planning and shared decision‐making processes may be useful.

Research has identified that goal planning is most effective when the goals are relevant to the person concerned, challenging but realistic and can be measured.^35^ It should be noted that many service users were experiencing co‐occurring health conditions and described barriers that impacted their experience of planning and achieving their goals. Pharmacists reported complexities in supporting service user‐identified goals and needing to adjust service user expectations to agree on realistic outcomes. However, service users largely identified positive outcomes from their goal planning and reported satisfaction with the goal planning process, highlighting that people may still achieve positive outcomes even when they do not achieve their end goal.^47^ Pharmacists also identified the importance of connecting service users to community agencies and resources, supporting outcomes when service user goals were outside their professional scope of practice. Pharmacists reported enjoying finding resources in their local communities and communicating and coordinating care with other health professionals. This finding underscores the importance of providing community pharmacists with the appropriate skills and training needed to support collaborative and effective goal planning, including how to advocate for those they are working with.^48^

This study must be viewed in the context of its strengths and limitations. The inclusion of goal‐planning experiences from both the perspective of pharmacists and service users strengthened this study, along with the diversity of pharmacy locations. However, participants were contacted after completing the *PharMIbridge* RCT, and their experiences may have differed from those who did not complete the intervention or participate in an interview. Additionally, pharmacists self‐selected to participate in the *PharMIbridge* RCT, and as such, may have different attitudes towards working with people experiencing SPMIs in comparison to community pharmacists more generally. While several strategies were employed to increase the trustworthiness of the research process (e.g., a single interviewer, the use of an interview guide, summary notes made at the completion of each interview, quality checking of transcriptions), a reflective approach to coding was undertaken which may have influenced intercoder reliability. Given this study's qualitative nature, we could not compare objective goal outcomes with goal planning experiences, which is a significant area for further investigation. This is particularly relevant to community pharmacy settings as there is limited research regarding goal‐planning outcomes.

Overall, the findings from this study offer a unique understanding of how participants experienced person‐centred goal‐planning processes. Not only were goals important in supporting behaviour change, but they also provided an opportunity for service users to learn about themselves and identify their health concerns. Equally, pharmacists were able to understand more about the health challenges and barriers faced by service users experiencing SPMIs. Both pharmacists and service users noted that a 6‐month intervention was not always adequate for longer‐term health‐related behaviour change, particularly for those experiencing complex health issues and needs. Flexibility in the provision of the intervention was seen as crucial, catering to service users at different stages of readiness and allowing for an individualised approach to service delivery.

## 5 CONCLUSION

This study provides further insights into the experience of person‐centred goal planning in community pharmacy settings from the perspectives of both service users experiencing SPMIs and pharmacists. As goal planning is increasingly incorporated in community pharmacy practice,^49^ results from this study indicate that the goal planning experience from both the pharmacist and service user perspective was largely positive. The findings from this study support the inclusion of goal‐planning processes when assisting service users experiencing SPMIs to improve their health. However, further research regarding the use of goal planning in primary health settings (e.g., community pharmacy) is needed, particularly in regard to tools, strategies or training that could support future goal planning interventions for both service users and pharmacists.

## AUTHOR CONTRIBUTIONS

Amanda J. Wheeler, Claire O'Reilly, Sara S. McMillan and Sarira El‐Den contributed to the design of the overall randomised controlled trial, and along with Victoria Stewart, Jie Hu and Jack C. Collins designed this particular study. Victoria Stewart collected the data and Sara S. McMillan and Victoria Stewart analysed the data. Victoria Stewart wrote the first draft of the manuscript. All authors commented on subsequent versions and read and approved the manuscript.

## CONFLICT OF INTEREST STATEMENT

The authors declare no conflict of interest.

## ETHICS STATEMENT

The protocol and pharmacist training program received Griffith University Human Research Ethics Committee approval (HREC/2019/473). Verbal consent was obtained from all participants before interview.

## Data Availability

The data sets generated during and/or analysed during the current study are not publicly available due to the sensitive nature of the results, but deidentified summaries of the data may be available from the corresponding author upon reasonable request.
